# MALDI-TOF MS as a new tool for the identification of *Dientamoeba fragilis*

**DOI:** 10.1186/s13071-017-2597-3

**Published:** 2018-01-04

**Authors:** Adriana Calderaro, Mirko Buttrini, Sara Montecchini, Sabina Rossi, Giovanna Piccolo, Maria Cristina Arcangeletti, Maria Cristina Medici, Carlo Chezzi, Flora De Conto

**Affiliations:** Department of Medicine and Surgery, University of Parma, Parma, Italy

**Keywords:** *Dientamoeba fragilis*, MALDI-TOF mass spectrometry, Identification

## Abstract

**Background:**

In this study for the first time, a *Dientamoeba fragilis* protein profile by MALDI-TOF MS was created in order to identify specific markers for the application of this technology in the laboratory diagnosis of dientamoebiasis. In particular, one *D. fragilis* reference strain was used to create a reference spectrum and 14 clinical isolates to verify the reliability of the obtained results.

**Results:**

While 15 peaks were found to be discriminating between the reference strain and the culture medium used, six peaks, observed in all the 14 strains tested, were considered as markers able to identify *D. fragilis*.

**Conclusions:**

In our hands, MALDI-TOF MS technology was demonstrated as a useful tool to be used in association with or in replacement of the real-time PCR assay for the identification of *D. fragilis* used in our laboratory on xenic cultures, due to its accuracy, rapidity and low cost.

**Electronic supplementary material:**

The online version of this article (10.1186/s13071-017-2597-3) contains supplementary material, which is available to authorized users.

## Background

The role of *Dientamoeba fragilis* as a causative agent of intestinal parasitosis has been long discussed in the scientific community; however, the evidence collected in recent years has led to re-evaluation of the pathogenicity of this protozoan. Dientamoebiasis has a cosmopolitan distribution and is found in a large number of patients with diarrhea, abdominal pain, flatulence, fatigue and loss of appetite in the absence of other enteric pathogens [1–4]. The global prevalence of *D. fragilis* infection ranges from 0.5 to 16% [5].

Until 2014, when cyst and precystic stages were described for the first time in clinical human specimens [6], only the trophozoite stage in stools of infected individuals was known. However, this recent finding is still considered preliminary, requiring further testing to validate the existence of these stages in human hosts [7].

Traditionally, the laboratory diagnosis of *D. fragilis* infection is performed by the microscopic examination of permanently stained fecal smears. However, this approach is difficult due to several factors, such as the discontinuous shedding of *D. fragilis* and the rapid degeneration of trophozoites [2, 3]. In addition to the expertise of the parasitologist performing microscopic examination, the success in detecting *D. fragilis* is positively influenced by the examination of multiple fecal samples, the use of suitable staining techniques and the use of culture, which has proven to be two times more sensitive than stained smears in detecting *D. fragilis* [2, 8–10]. Because of the cited difficulties, few laboratories routinely test for *D. fragilis*, and few prevalence data, probably underestimated, are available [1, 2, 11, 12]. At present, the availability of amplification assays (such as real-time PCR) targeting the genes encoding for ribosomal RNA allows a more rapid and sensitive laboratory diagnosis, despite higher costs [12].

The matrix-assisted laser desorption ionization time-of-flight mass spectrometry (MALDI-TOF MS) has already revolutionized the identification of bacteria and fungi for diagnostic purposes due to its high resolution and low cost for single determination, ranking as a valid alternative to the biochemical and molecular conventional identification systems [13]. Nevertheless, this technology has not yet been used for the routine identification of intestinal protozoa. However, in recent years, different research groups have performed studies for the identification of intestinal protozoa either by detecting specific biomarkers as in the case of *Cryptosporidium* spp. [14], *Giardia* spp. [15] and *Entamoeba histolytica*/*Entamoeba dispar* [16], or by creating a specific protein profile as in the case of *Blastocystis hominis* [17] and *Trichomonas vaginalis* [18].

The aim of this study was the creation by MALDI-TOF MS of a *D. fragilis* protein profile, which is not yet available in the commercial database. Moreover, we evaluated the use of this technology in the laboratory diagnosis of dientamoebiasis for a possible association with or replacement of the currently used PCR-based assay, which is expensive and requires well-trained personnel.

## Methods

### *Dientamoeba fragilis* Strains

In this study the *D. fragilis* No. 3313 (DF3313), previously characterized by real-time PCR and sequencing [9], was used as reference strain, and 14 *D. fragilis* strains (Nos. 32, 64, 82, 121, 130, 357, 372, 471, 1272, 1668, 1686, 1710, 1719, 2005), previously isolated from fecal samples in our laboratory and identified by real-time PCR [9], were included. The reference strain and the clinical isolates were cultivated in Robinson’s medium, as previously described [9, 19]; these strains currently belong to our collection and they are used in our laboratory for research. The reference strain was used to create a reference spectrum by MALDI-TOF MS and the clinical isolates were used to verify the reliability of the obtained results. Protein extraction of each strain was performed as previously described [16].

For the reference strain two independent experiments using 2 independent cultures on 2 different days by 2 independent operators were run (inter-assay reproducibility) and 6 replicates/run were analyzed in order to ensure the reproducibility of the results obtained (intra-assay reproducibility).

### Detection limit of MALDI-TOF MS for *D. fragilis*

Aliquots of 1 ml from serial ten-fold dilutions (from 10^6^ to 10^3^ trophozoites/ml) of the DF3313 strain [9] cultured in Robinson’s medium were subjected to protein extraction, as previously described [16], and to the MALDI-TOF MS analysis. Each dilution was prepared using the liquid phase of the Robinson’s medium.

### Experimentally seeded samples

Five hundred microliters of two *D. fragilis* cultures (DF3313 and No. 1686), each containing 10^6^ trophozoites/ml, were mixed with an equal volume of sterile culture medium added with 1 g of human feces previously assessed negative for *D. fragilis* by real-time PCR [9]. An aliquot of 1 ml of this suspension was centrifuged at 3000× *g* for 10 min and the pellet obtained was subjected to protein extraction and to the MALDI-TOF MS analysis.

### MALDI-TOF MS: Spectra acquisition

Proteic extracts were analyzed by MicroFlex LT mass spectrometer (Bruker Daltonics, Bremen, Germany); spectra were acquired using MBT_Standard method (positive linear mode, laser frequency 60 Hz, ion source voltage 20 kV, mass range 2–20 kDa) in manual mode acquisition with at least overall 240 laser-shots, in order to obtain a clear signal with an intensity > 10^4^ arbitrary units, by 40 shot steps discarding those with an intensity < 10^3^ arbitrary units. Each shot step was made in different points of the well with a variable laser intensity ranging from 30 to 50% for each single shot step. Six replicates/run for each experiment were analyzed.

In order to minimize the variability associated with technical or biological parameters, the experiments were performed under controlled cultivation and sample preparation conditions and consistent technical configurations, assuring a high repeatability and reproducibility between experiments. In each experiment, the “Bacterial Test Standard” (Bruker Daltonics) for calibration was used according to the manufacturer’s instructions.

### Spectra analysis

For all the spectra obtained by MALDI-TOF MS manual acquisition, “Smoothing” and “Baseline” were performed using Flex Analysis software (version 3.3 Bruker Daltonics). The replicates with a profile significantly different from the others were eliminated. In order to select the peaks differentiating *D. fragilis* from Robinson’s medium all of the replicates, obtained in the two independent experiments, were imported into ClinProTools statistical software (version 2.2, Bruker Daltonics) and automatically recalibrated [20]. Unsupervised statistical testing of the datasets was performed on the basis of principal components analysis (PCA) to visualize the homogeneity and heterogeneity of the protein spectra and the results were displayed in a three-dimensional score plot generated by the software. PCA reduces the variability of the complex datasets, automatically generating a set of new variables called the principal component (PC). Moreover, the software was used to identify peaks with a statistically significant difference between the *D. fragilis* reference strain and Robinson’s medium by comparison of the two average spectra automatically created from the replicates of the strain or of the Robinson’s medium. From all peaks, ClinProTools derives some characteristics such as the peak area/intensity, which are considered as features and used for the further processing. The peak area/intensity value, together with the values obtained from other features, were automatically analyzed by statistical tests (in this study by the analysis of variance test - ANOVA) included in the software to calculate the *P*-value. The *P*-value obtained provides a measure of the probability of the strength of an association/dissociation among the different specific peaks for the classes analyzed. Differences were considered significant when *P* < 0.05; however, in this study, only peaks with a *P* < 0.0001 were considered.

To assess the reliability of the discriminating peaks, the analysis of the spectra was performed by ClinProTools software on those obtained from the DF3313 strain dilutions used for the detection limit, from the 14 clinical isolates and from the two experimentally seeded fecal samples. The presence/absence of each discriminating peak was evaluated in comparison to the average spectrum automatically created from each replicate. All ClinProTools analyses were performed in the mass range 3000–11,000 Da, with a signal to noise ratio (S/N) value of 5, and a threshold value of 0.2.

## Results

**Fig. 1:**
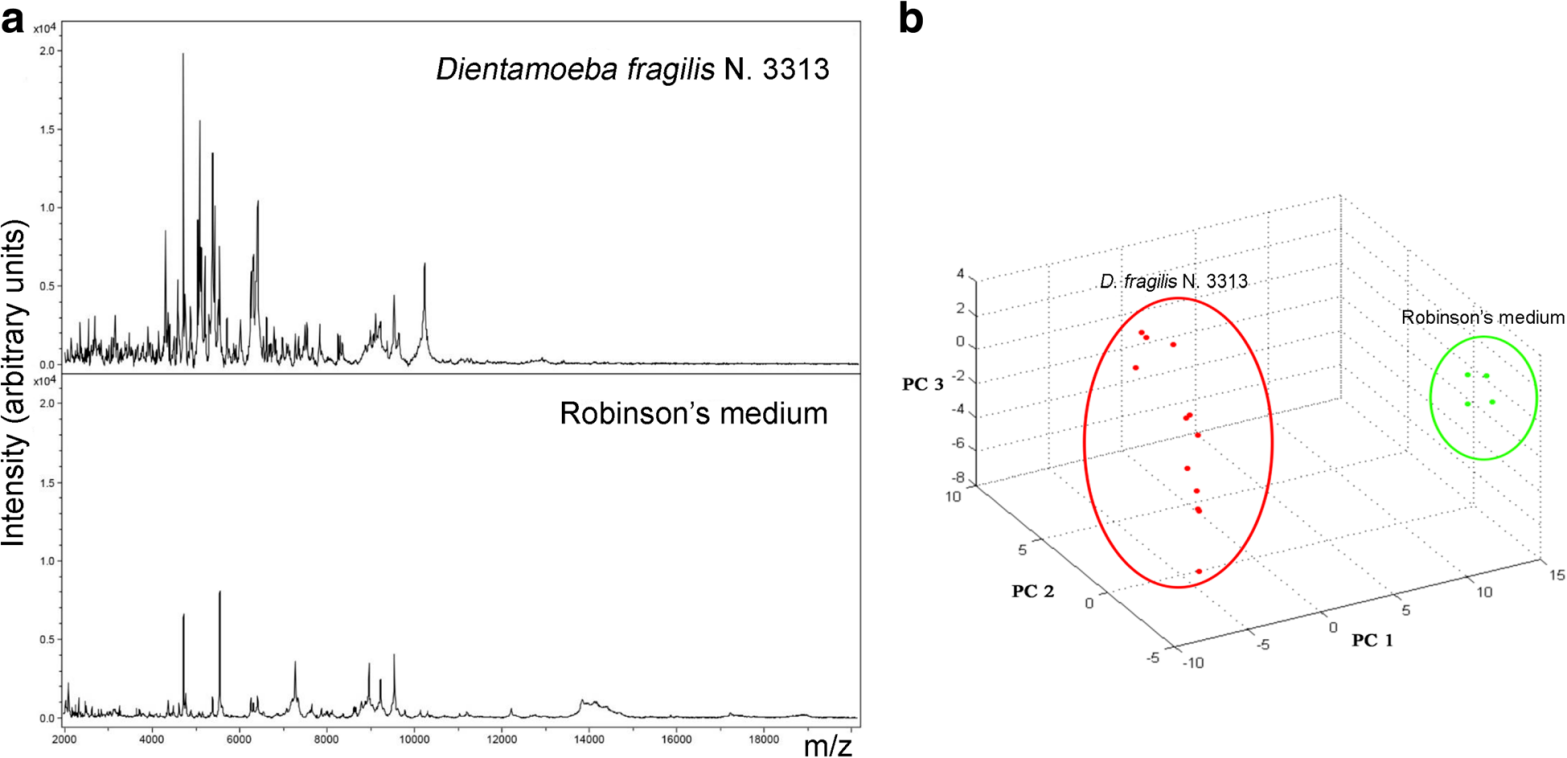
Comparison of the spectra obtained for the *D. fragilis* No. 3313 reference strain (DF3313) and the Robinson’s medium alone (**a**) and cluster analysis by principal components analysis (PCA) (**b**) of the replicates of the DF3313 (*red*) and of the Robinson’s medium alone (*green*)

The analysis of the DF3313 reference strain by MALDI-TOF MS showed a reproducible protein profile in the replicates obtained both in the individual experiments (intra-assay reproducibility) and in the two different experiments performed on two different days (inter-assay reproducibility) (Additional file 1: Figure S1). From all these spectra, the average reference spectrum was created. When the same analysis was performed on the Robinson’s medium alone, the average spectrum showed the presence of some peaks overlapping those found in the protein profile of the DF3313 reference strain (Fig. 1a). The PCA of the replicates of the DF3313 reference strain and of those of the Robinson’s medium, by using statistical software, showed two completely separated clusters (Fig. 1b).

The same analysis showed the absence of peaks in the range 11,000–20,000 Da, and a signal to noise ratio with a low value in the range 2000–3000 Da, leading to the exclusion of these ranges for subsequent analyses, performed exclusively for the range 3000–11,000 Da (Additional file 2: Figure S2).

**Fig. 2:**
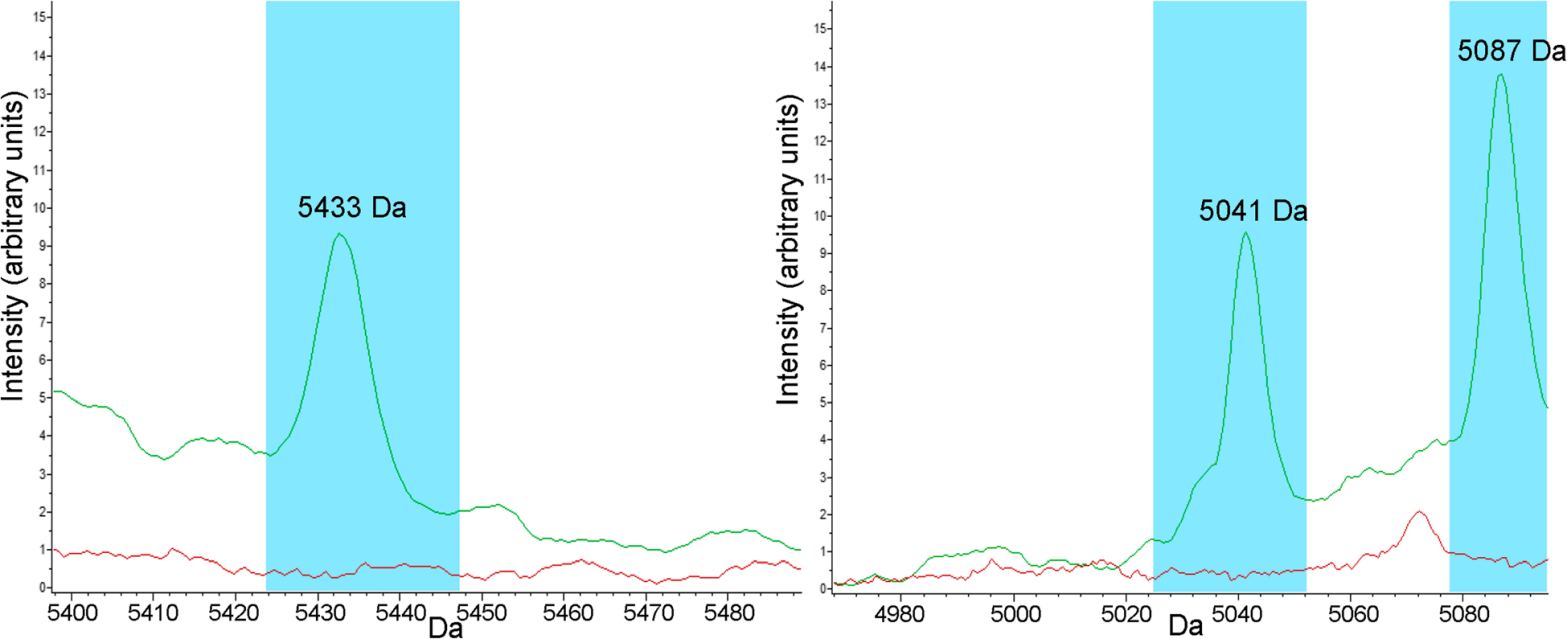
Examples of peaks (5433, 5041 and 5087 Da) discriminating the *D. fragilis* No. 3313 reference strain (*green*) from the Robinson’s medium alone (*red*) on the basis of the average spectra obtained

The statistical analysis performed for the range 3000–11,000 Da showed the presence of 19 peaks enabling to differentiate between *D. fragilis* and Robinson’s medium (Table 1, Fig. 2). Among the 19 peaks, 15 belonged to *D. fragilis* and 4 to Robinson’s medium; these latter peaks (7277, 9229, 9539 and 6415 Da) were excluded from further analysis.

**Table 1:** List of the 19 discriminating peaks found by statistical analysis and detection limit of each peak. All *P*-values < 0.0001

No.	m/z	Rm	DF	Detection limit (trophozoites/ml)
		Mean ± SD	Mean ± SD	
1	5100	1.37 ± 0.14	6.80 ± 0.54	10^3^
2	7277	13.91 ± 0.37	2.39 ± 0.87	–
3	9229	8.94 ± 0.26	3.19 ± 0.85	–
4	6404	3.08 ± 0.11	12.06 ± 1.18	10^6^
5	9539	14.63 ± 0.65	4.87 ± 1.85	–
6	5205	0.56 ± 0.08	8.20 ± 1.29	10^6^
7	6377	2.23 ± 0.14	6.51 ± 0.87	10^6^
8	6387	2.76 ± 0.17	6.49 ± 0.80	10^6^
9	4758	3.70 ± 0.23	7.22 ± 0.80	10^6^
10	5041	0.88 ± 0.10	9.94 ± 2.13	10^5^
11	6415	5.23 ± 0.23	11.85 ± 1.96	–
12	4309	0.96 ± 0.10	8.61 ± 2.28	10^5^
13	6285	1.95 ± 0.11	5.87 ± 1.27	10^6^
14	5121	0.68 ± 0.05	9.10 ± 2.81	10^6^
15	5516	2.94 ± 0.29	5.11 + 0.60	10^6^
16	10,239	0.76 ± 0.06	7.37 + 2.46	10^6^
17	5087	1.12 ± 0.02	14.26 ± 5.92	10^5^
18	5433	0.95 ± 0.11	9.57 ± 4.46	10^6^
19	6298	2.27 ± 0.13	6.07 ± 2.04	10^6^

*Abbreviations*: Mean, the peak area/intensity average; Rm, Robinson’s medium; DF, *Dientamoeba fragilis*; SD, standard deviation

**Fig. 3:**
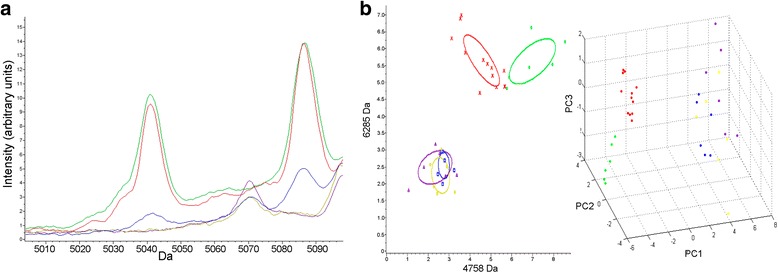
Analysis of the spectra obtained for the *D. fragilis* No. 3313 reference strain at different trophozoite concentrations (10^6^ trophozoites/ml in *green*, 10^5^ trophozoites/ml in *blue*, 10^4^ trophozoites/ml in *yellow*, 10^3^ trophozoites/ml in *purple*) in comparison to the average reference spectrum in red. **a** Examples of the presence/absence of two discriminating peaks (5041 and 5087 Da). **b** Cluster analysis by “2D distribution view” of the first two best separating peaks (left) (the ellipses represent the standard deviation of each concentration average of the peak area/intensity) and by principal components analysis (PCA) (right)

The detection limit of the MALDI-TOF MS for the detection of each peak was 10^6^ trophozoites/ml for 11 of the 15 peaks, 10^5^ trophozoites/ml for 3 peaks (5041, 5087 and 4309 Da), and 10^3^ trophozoites/ml for the remaining peak (5100 Da) (Table 1, Fig. 3a). The PCA performed on the spectra obtained for each trophozoite concentration tested in the experiment to assess the detection limit showed that those obtained from the DF3313 strain at the 10^6^ trophozoites/ml concentration were completely separated from those at the 10^5^–10^3^ trophozoites/ml concentrations, whilst being close to those obtained in the inter- and intra-assay reproducibility (Fig. 3b).

To verify the reliability of the 15 peaks as *D. fragilis* markers, the MALDI-TOF MS analysis was extended to the 14 clinical isolates. The results of this analysis are shown in Table 2. In particular, the peak at 10,239 Da was not found in any of the clinical isolates, while the peaks at 4309, 4758, 5041, 5100, 5516 and 6387 Da were found in all the 14 (100%) clinical isolates. These six peaks were considered discriminating peaks for *D. fragilis*.

**Table 2:** Presence/absence of the 15 *D. fragilis* discriminating peaks in the 14 clinical isolates tested

Strain	Peak mass (Da)														
	4309	4758	5041	5087	5100	5121	5205	5433	5516	6285	6298	6377	6387	6404	10,239
357	+	+	+	–	+	+	+	+	+	–	+	+	+	+	–
372	+	+	+	–	+	+	+	–	+	–	+	–	+	+	–
471	+	+	+	–	+	+	–	–	+	–	–	–	+	–	–
1272	+	+	+	–	+	+	+	–	+	–	+	+	+	+	–
1668	+	+	+	–	+	–	+	+	+	–	+	+	+	+	–
1686	+	+	+	–	+	–	–	–	+	+	–	+	+	–	–
1710	+	+	+	–	+	–	–	–	+	–	–	+	+	–	–
1719	+	+	+	–	+	–	+	+	+	–	+	+	+	+	–
2005	+	+	+	–	+	–	+	+	+	–	+	+	+	+	–
64	+	+	+	–	+	+	–	–	+	–	+	+	+	–	–
32	+	+	+	+	+	–	+	+	+	–	+	+	+	+	–
82	+	+	+	–	+	+	–	–	+	+	+	+	+	–	–
121	+	+	+	–	+	–	+	+	+	+	+	+	+	+	–
130	+	+	+	+	+	–	+	+	+	+	+	+	+	+	–
%	100	100	100	14.3	100	42.9	71.4	50.0	100	26.7	78.6	85.7	100	71.4	0

*Abbreviations*: Da, Dalton; +, presence; –, absence

**Fig. 4:**
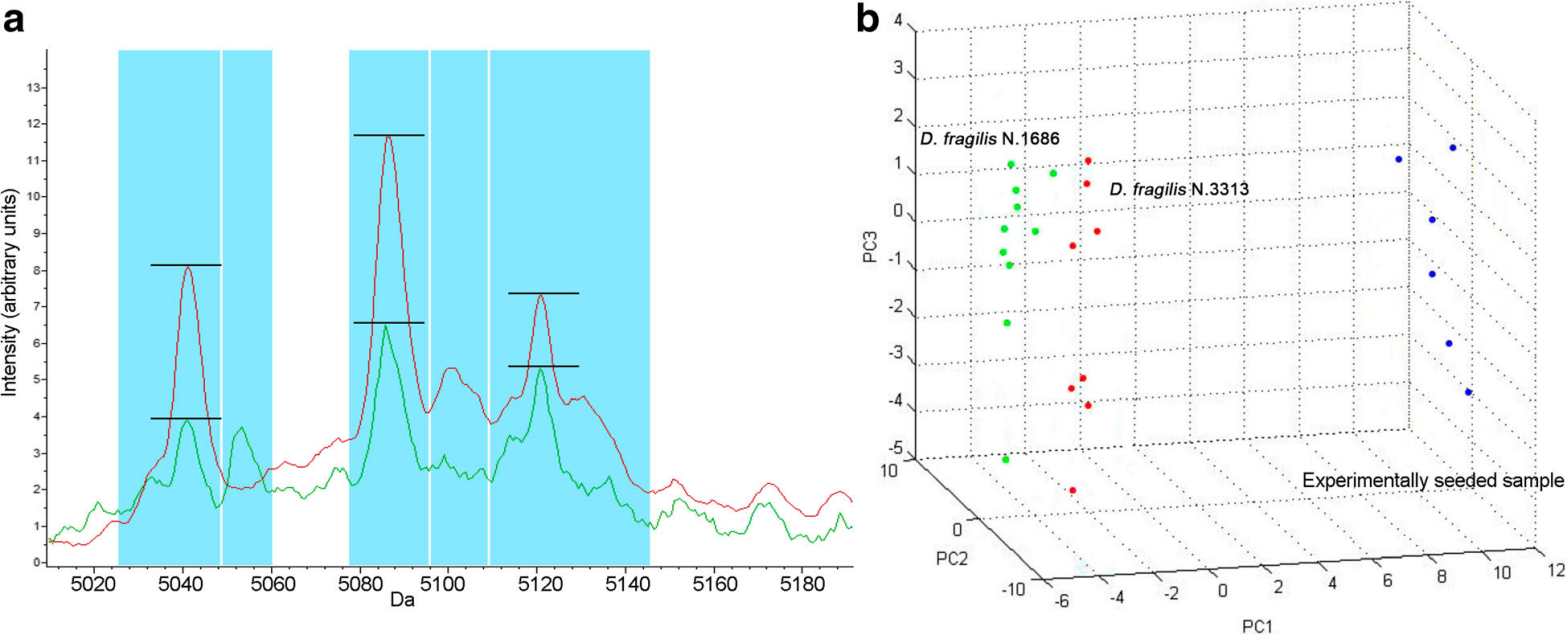
Analysis of the spectra obtained from the two *D. fragilis* experimentally seeded fecal samples. **a** Example of discriminating peaks found in the fecal sample experimentally seeded with the *D. fragilis* No. 3313 reference strain at the 10^6^ trophozoites/ml concentration (*green*) in comparison to the average reference spectrum (*red*). **b** Cluster analysis by principal components analysis (PCA) of the fecal sample experimentally seeded with the *D. fragilis* No. 1686 clinical isolate at the 10^6^ trophozoites/ml concentration (*blue*) in comparison to the *D. fragilis* No.1686 strain at the same concentration without feces (*green*) and to the average reference spectrum (*red*)

The analysis of the fecal sample experimentally seeded with the DF3313 reference strain showed the presence of 7 out of the 15 peaks found in the same strain when tested without feces (Table 3). Among these 7 peaks, only 2 (4309 and 5041 Da) were included in the 6 peaks considered discriminating for *D. fragilis*. Similarly, in the fecal sample experimentally seeded with the *D. fragilis* No. 1686 clinical isolate, 4 out of the 8 peaks found in the same strain when tested without feces were detected and of these only 3 (4309, 4758 and 5100 Da) were included among the 6 *D. fragilis* discriminating peaks (Table 4). The intensity of these peaks was lower than that found for the same peaks detected in the same strains tested in the absence of fecal material (Tables 3 and 4, Fig. 4a). The statistical analysis performed by PCA on the replicates of the fecal sample experimentally seeded with the *D. fragilis* No. 1686 clinical isolate and the replicates of the same strain in the absence of fecal material in comparison to those obtained for the DF3313 reference strain showed 2 completely separated clusters: the first one included the DF3313 reference strain and the *D. fragilis* No. 1686 in the absence of fecal material, and the second one included only the experimentally seeded stool sample (Fig. 4b).

**Table 3:** Presence/absence of peaks of the *D. fragilis* No. 3313 reference strain under different conditions

Mass (Da)	*D. fragilis* No. 3313	FSES1
	Mean ± SD	Mean ± SD
4309	8.61 ± 1.78	8.49 ± 1.37
4758	7.22 ± 0.80	–
5041	9.94 ± 1.63	4.49 ± 0.58
5087	14.26 ± 4.80	7.11 ± 0.84
5100	6.80 ± 0.54	–
5121	9.10 ± 2.28	5.88 ± 1.12
5205	8.20 ± 0.97	4.98 ± 0.57
5433	9.57 ± 3.64	12.35 ± 1.58
5516	5.11 ± 0.60	–
6285	5.87 ± 1.27	–
6298	6.07 ± 2.04	–
6377	6.51 ± 0.87	–
6387	6.49 ± 0.80	–
6404	12.06 ± 1.18	–
10,239	7.37 ± 2.00	3.24 ± 0.74

*Abbreviations*: Da, Dalton; Mean, the peak area/intensity average; SD, standard deviation; DF3313, *D. fragilis* No. 3313 reference strain; FSES1, fecal sample experimentally seeded with the *D. fragilis* No. 3313 reference strain; –, peak not found

**Table 4:** Presence/absence of the peaks of the *D. fragilis* No. 1686 clinical isolate under different conditions

Mass (Da)	*D. fragilis* No. 1686	FSES2
	Mean ± SD	Mean ± SD
4309	3.86 ± 0.62	3.19 ± 0.84
4758	4.92 ± 1.20	3.46 ± 0.23
5041	4.81 ± 1.63	–
5100	3.48 ± 0.43	3.29 ± 0.64
5516	4.23 ± 0.43	–
6285	3.56 ± 0.32	2.36 ± 0.31
6377	6.90 ± 0.64	–
6387	7.40 ± 0.59	–

*Abbreviations*: Da, Dalton; Mean, the peak area/intensity average; SD, standard deviation; FSES2, fecal sample experimentally seeded with the *D. fragilis* No. 1686 clinical isolate; −, peak not found

## Discussion

MALDI-TOF MS technology can be used for the identification of microorganisms both by a specific protein profile and by the identification of specific protein markers [13, 16]. MALDI-TOF MS allows bacterial identification on the basis of the recognition of protein peak patterns which are characteristic and mostly constant for different bacterial species and is accomplished by pattern analysis of the mass spectra using mathematical tools. The technique is very rapid and only minimal amounts of bacteria are needed [21]. Conversely, parasite identification by a specific protein profile using MALDI-TOF MS had limited application [16]. The use of complex liquid media, such as that used for the cultivation of intestinal protozoa, interferes with the creation of a species-specific protein profile in contrast to what is normally done for bacteria and fungi, which grow on solid and axenic media. Nevertheless, the inter- and intra-assay reproducibility observed in the study has enabled the creation of a specific *D. fragilis* protein profile, although it was not possible to completely exclude peaks related to Robinson’s media supplemented with *Escherichia coli* as also observed for other intestinal parasites (*Entamoeba histolytica* and *Entamoeba dispar*) cultivated in the same medium [16]. For this reason, in this study the detection of *D. fragilis* was for the first time performed by MALDI-TOF MS through the recognition of specific protein markers.

The comparison between the spectrum of *D. fragilis* and that of Robinson’s medium alone has allowed the identification of 15 peaks (*P* < 0.0001) referring only to this protozoan.

The experiment to assess the detection limit of the different peaks showed that all 15 peaks, except four (4309, 5041, 5087 and 5100 Da), could only be detected by analyzing a high concentration of trophozoites/ml (10^6^). Although the MALDI-TOF MS analytical sensitivity observed in this study is analogous to that found for other parasites [16, 17], it cannot be excluded that the described peaks could be related to proteins with a low expression. This hypothesis has not been yet verified since the mass of the 15 peaks did not correspond to any molecular weight of the few *D. fragilis* proteins deposited in GenBank [22]. It is likely that when all the *D. fragilis* protein sequences become available, we will be able to detect those with a molecular weight similar to that of the peaks detected.

The reliability of the 15 peaks was evaluated by analyzing 14 clinical isolates. Only six peaks (4308, 4758, 5041, 5100, 5516 and 6387 Da) were found in all the tested strains, while nine were alternatively detected. These six peaks were taken into account as markers able to identify *D. fragilis*. It is noteworthy that four out of these six peaks were the same as those revealed at the lowest concentration (5041, 5087, 4309 Da at 10^5^ trophozoites/ml and 5100 Da at 10^3^ trophozoites/ml) in the experiment to assess the detection limit. For the remaining nine peaks detected in the reference strain and alternatively found in the tested clinical isolates, it could be hypothesized that the corresponding proteins could be dependent on the specific characteristics of the different strains or could be expressed in different conditions.

The analysis of experimentally seeded samples has shown that the fecal material can interfere with the detection of specific proteins, as previously described [16]. Despite the fact that in the present study some discriminating peaks were found, the intensity of these peaks was lower than that observed in the absence of fecal material. This result is not unexpected since, as previously reported, several parameters can affect the MALDI-TOF MS identification quality from clinical samples, such as the pathogen concentration, the presence of other microorganisms and the nature of the sample [13]. The development of an efficient and standardized pre-processing protocol to discard interfering substances is required to allow for the direct detection of *D. fragilis* from feces. For this reason, the identification of *D. fragilis* by MALDI-TOF MS was performed in this study after a culture step.

In our laboratory, the diagnosis of dientamoebiasis is performed on multiple fecal samples by microscopic examination of fresh and concentrated feces, according to standard procedures [9, 23], and cultivation in Robinson’s medium [9]. A real-time PCR assay targeting the 5.8S rRNA gene of *D. fragilis* is performed when trophozoites resembling this protozoan are observed [9]. The amplification of *D. fragilis* DNA fragments either by conventional PCR or by real-time PCR is directly applicable on fecal samples and has proven to be a sensitive and specific method for the diagnosis of dientamoebiasis, circumventing the insensitivity of microscopy or of culture-based diagnosis [9, 24]. However, these molecular methods remain cumbersome and, particularly with regard to the real-time PCR, expensive.

Taking into account that cultivation in xenic medium is a fundamental step in parasitic diagnosis in order to reveal the presence of different parasites, the advantages of MALDI-TOF MS are evident, particularly in terms of rapidity, simplicity and cost saving. Despite the high instrument cost, the cost saving is achieved as its use is not limited to the diagnosis of dientamoebiasis alone; in fact, the use of MALDI-TOF MS is constantly increasing in the microbiology laboratories for identification of other microbial strains and so the cost would be spread across a variety of activities [25].

In this study, MALDI-TOF MS was successfully applied for the first time in order to replace the PCR assay for the identification of *D. fragilis* strains isolated from clinical samples. MALDI-TOF MS could also be performed to avoid the use of permanent staining, suffering of the variability in size and shape of the protozoans [26] and of the poor sensitivity when compared to culture in Robinson’s medium [2].

## Conclusions

The MALDI-TOF MS technology for the identification of *D. fragilis* demonstrated to be a valid alternative to the Real-time PCR assay used in our laboratory on xenic cultures, being accurate, more rapid, and easy to use, particularly concerning the protein extraction. These features, together with the lower costs observed in our experience (0.50 € *vs* 100 € per determination), rank this technology as a valid tool for the routine diagnosis of dientamoebiasis.

## Additional files

Additional file 1: Figure S1. Spectra obtained (six replicates/run) for the *D. fragilis* No. 3313 reference strain in the two different experiments. (TIFF 447 kb)

Additional file 2: Figure S2. Average spectra of the *D. fragilis* No. 3313 reference strain in the range 2000–20,000 Da. In the box, magnification of the range 3500–11,000 Da used in this study for all analyses performed. (TIFF 274 kb)

## Abbreviations

ANOVA: analysis of variance
Da: Dalton
DF3313: *D. fragilis* No. 3313 reference strain
MALDI-TOF MS: matrix-assisted laser desorption ionization time-of-flight mass spectrometry
PCA: principal components analysis
PCR: polymerase chain reaction
