# Supplementary figures and images for: MALDI-TOF MS as a new tool for the identification of Dientamoeba fragilis
Source: Parasit Vectors. 2018 Jan 4;11:11. doi: 10.1186/s13071-017-2597-3 (PMC5755284; doi:10.1186/s13071-017-2597-3)

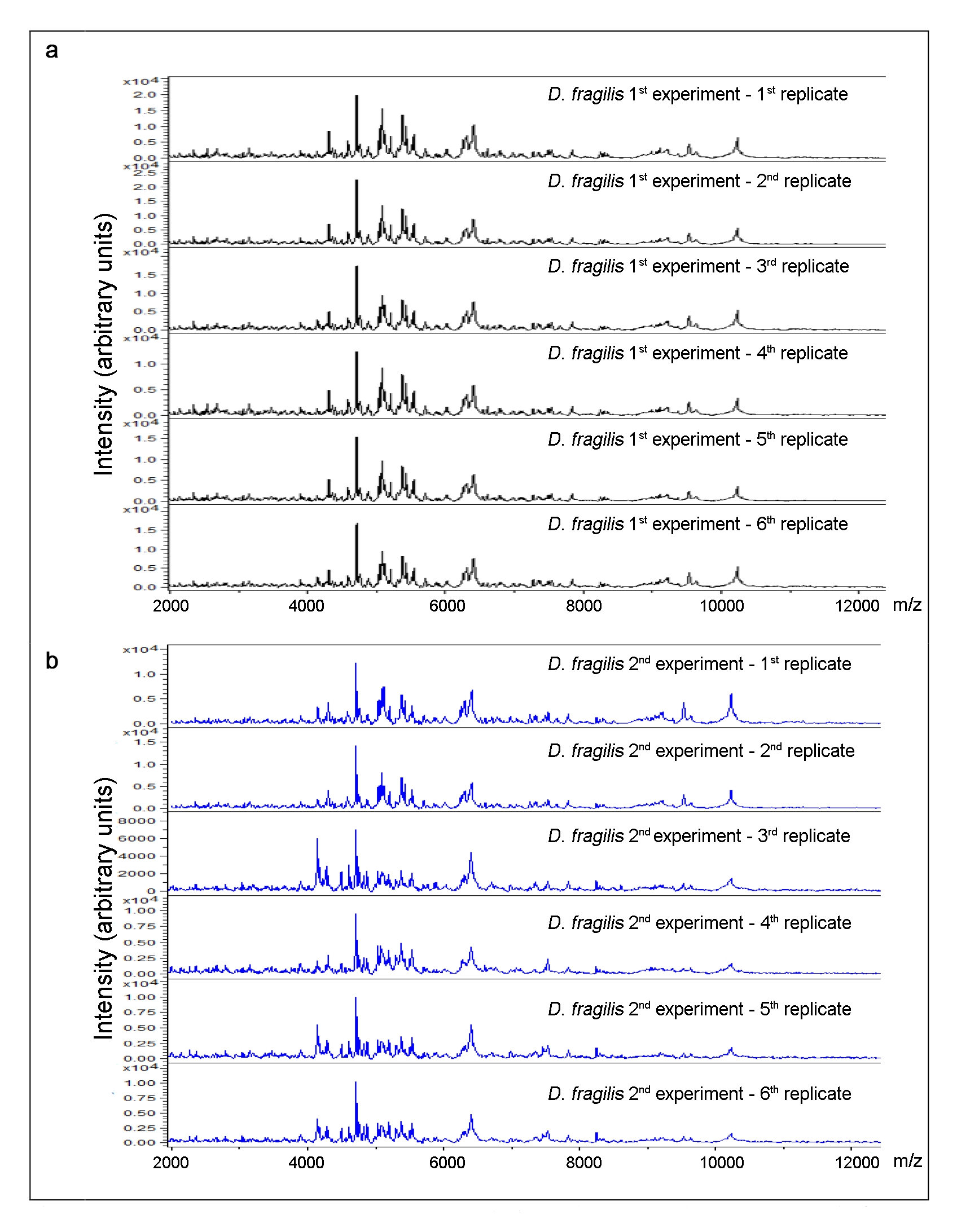

Supplement: Supplementary file 1 — Spectra obtained (six replicates/run) for the D. fragilis No. 3313 reference strain in the two different experiments. (TIFF 447 kb) [file 13071_2017_2597_MOESM1_ESM.tif]

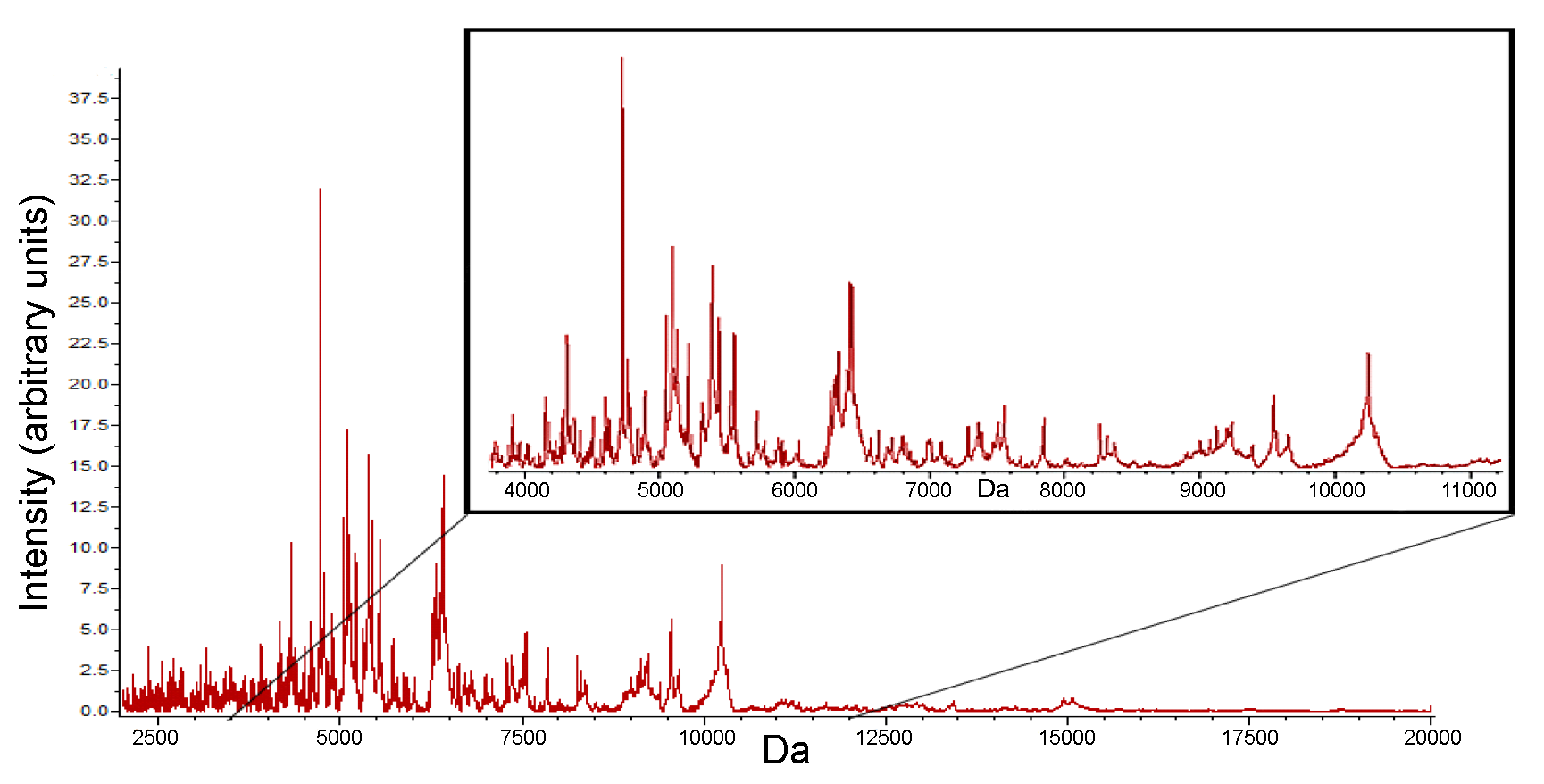

Supplement: Supplementary file 2 — Average spectra of the D. fragilis No. 3313 reference strain in the range 2000–20,000 Da. In the box, magnification of the range 3500–11,000 Da used in this study for all analyses performed. (TIFF 274 kb) [file 13071_2017_2597_MOESM2_ESM.tif]
